# Body-worn cameras to prevent workplace aggression among ticket inspectors: Protocol for a randomized controlled trial

**DOI:** 10.1371/journal.pone.0342270

**Published:** 2026-02-03

**Authors:** Camilla Bank Friis, Merlin Schaeffer, Juliane Starcke Neergaard, Lasse Suonperä Liebst

**Affiliations:** 1 Department of Society and Politics, Aalborg University, Copenhagen, Denmark; 2 Department of Sociology, University of Copenhagen, Copenhagen, Denmark; 3 WZB Berlin Social Science Center, Berlin, Germany; 4 The Netherlands Institute for the Study of Crime and Law Enforcement (NSCR), Amsterdam, The Netherlands; Université Paris Dauphine: Universite Paris Dauphine, FRANCE

## Abstract

**Background:**

Service and frontline personnel are among the occupational groups with the highest rates of workplace aggression. To address the risk of victimization, various preventive measures have been introduced, with body-worn cameras increasingly adopted. However, evidence of their effectiveness remains inconclusive, with existing studies heavily concentrated in U.S. policing contexts, limiting the generalizability of findings across settings. This study aims to strengthen the evidence base through a randomized controlled trial testing the preventive effect of body-worn cameras among ticket inspectors in Denmark.

**Methods:**

The trial will involve approximately 60 inspectors employed by three Danish public transport companies. Randomization will occur at the shift level, yielding approximately 3,000 shifts in total. The main analysis compares wearing a camera versus not wearing a camera, pooling data from all three companies. A secondary analysis, restricted to two companies, additionally tests whether a visible badge notifying passengers of potential recording strengthens any preventive effect. In parallel, field observations of inspection workdays will be conducted to gain in-depth insights into how and why cameras may influence interactions.

**Discussion:**

This study presents a rare randomized controlled trial on the preventive effect of body-worn cameras against workplace aggression outside a U.S. policing context. If the hypothesis of a preventive effect is confirmed, the findings will have direct practical implications for deploying this technology to reduce workplace aggression.

## Introduction

Work-related violence and victimization present significant challenges for society, employers, and workers. Service and frontline personnel, in particular, face a heightened risk of harassment, threats, and violence compared with other occupational groups [1]. Such vulnerability can result in physical, psychosocial and socioeconomic consequences, including absenteeism, reduced work capacity, and impaired mental well-being [2]. In response, substantial political and organizational efforts have been undertaken to mitigate these risks, such as public campaigns, conflict management training, and improved documentation of incidents.

In recent years, body-worn cameras have emerged as a preventive technology to manage difficult face-to-face encounters with citizens, clients, and customers that service and frontline staff face. The number of occupational groups where body-worn cameras have been used or tested is expanding, and includes police officers [3], transit workers [4], prison and probation service staff [5], retail workers [6], ambulance crews [7], and healthcare professionals [8].

Despite this growing interest, empirical evidence on the effectiveness of body-worn cameras remains inconclusive [9]. Prior research indicates that their effects are highly context-dependent—potentially reducing, increasing, or having no impact on work-related victimization. Moreover, most studies have been conducted with U.S. police, whose experiences differ substantially from those of other frontline workers in other national contexts. In sum, there is a clear need to test the preventive effect of body-worn cameras in settings beyond U.S. policing.

The current study protocol outlines a randomized controlled trial of body-worn cameras that seeks to test whether wearing a camera on the uniform reduces victimization risk, using a design that advances the existing literature in several respects. We conduct a large-scale trial to help resolve the ongoing uncertainty over whether body-worn cameras have a robust effect at all. This test is conducted outside the U.S. policing context—specifically with Danish ticket inspectors—to help clarify the literature’s uncertainty regarding the context dependency of effects. Additionally, we incorporate a range of methodological strategies in our trial design that offer advantages over existing studies. Below, we review the current literature and its limitations and, on this basis, present the study protocol developed to address these gaps.

### Randomized controlled trials on body-worn camera effects

The development of body-worn cameras has led to a growing number of international studies examining their impact on frontline staff’s interactions with citizens, particularly in U.S.-based police–public encounters [10,11]. These studies have explored the camera’s effect on police use of force [12], number of arrests [13], citizen complaints [14], and officer exposure to violence [15]. While studies have looked beyond the policing context, these studies have typically not implemented randomized controlled trials to test the effect of cameras among groups such as prison and probation service staff [5] or ambulance crews [7].

Theoretically, cameras are expected to regulate behavior through a “deterrence effect” or “threat mechanism,” in which their presence signals a higher likelihood of apprehension or conviction, thereby encouraging behavioral compliance [16]. Further, cameras are assumed to promote self-awareness and socially desirable behavior, influencing both the person carrying the camera and the citizen [17].

Beyond deterrence and self-awareness, the camera’s effect may also operate through alternative mechanisms, such as perceptions of procedural justice or organizational practices of conflict management. For example, one study found that citizens reported higher perceived procedural justice in encounters with officers carrying a body-worn camera than in those without [18], which in turn could reduce citizen aggression and staff victimization risk. Additionally, attending to alternative pathways is important if cameras increase rather than decrease victimization. Such a finding could suggest that cameras are experienced as instruments of power display, potentially eliciting defensive or resistant responses from citizens and escalating interactions [19,20].

In line with these considerations, intervention studies examining whether cameras have a preventive effect on victimization of police officers present mixed results. Some U.S.-based studies report an increase in assaults on officers wearing cameras [3], while others from the U.S. and Australia find no significant effect of cameras on victimization risks [15,21]. A meta-analysis concludes significant variation in results regarding assaults against officers, suggesting that cameras can have different effects on citizen behavior under different circumstances. However, these contextual heterogeneities have not yet been documented or understood [9], leaving the interpretation of these mixed results unclear.

A rare exception from the U.S.-police context is a study by Ariel and colleagues from England and Wales that examined assaults against transport personnel [4]. Using 33 train stations as the unit of analysis, they reported a substantial reduction in official assault reports, suggesting that body-worn cameras may help prevent workplace victimization. Although noteworthy for its non-police setting, the study has a key limitation that our trial seeks to address: despite efforts to minimize spillover effects from camera-wearing officers to their colleagues without cameras, randomizing only 33 stations is unlikely to achieve a true balance of confounding factors between treatment and comparison groups. To overcome this, our design randomizes at the level of each inspector’s work shifts, rather than by location alone.

This limitation is closely linked to other methodological issues that call into question the validity of the current evidence and that our study seeks to address. Frequently—also in the study by Ariel and colleagues—victimization is measured using officially recorded incidents [see, e.g., 15]. This approach tends to bias findings toward serious and violent incidents, while overlooking the many more subtle and verbal forms of victimization (e.g., offensive remarks, verbal threats) that often go unreported, despite their potential to reduce well-being. Measuring these more subtle and verbal forms of victimization—as we will do with a survey instrument—is crucial, as inspectors are more often exposed to harassment and verbal threats than to physical violence [1]. The use of a survey instrument in our design also offers an advantage over prior work by enabling us to estimate both intention-to-treat and treatment-on-the-treated effects. Specifically, we survey each treated unit to determine whether a ticket inspector actually followed protocol and wore the camera as instructed (intention-to-treat) or not (actual treatment).

A further methodological limitation is that the experimental literature offers few insights into the effect of body-worn cameras on staff’s perceived safety—an outcome that can, and in our study will, be measured using the survey instrument. The few studies that have considered such perceptions report conflicting views on the camera’s potential to enhance safety across employee groups [22–25]. However, this existing research is based on expectations or retrospective assessments rather than real-time experiences of safety while wearing the camera.

A final methodological limitation of the literature concerns the scarcity of studies examining what happens during actual interactions involving cameras. Although deterrence is a dominant explanation, prior research has not directly investigated the in situ mechanisms at work—for example, whether staff behave differently when wearing a camera. As a result, little is known about the situational mechanisms that may come into play when frontline staff introduce technological surveillance tools into their interactions with citizens [26]. We will address this limitation by conducting a field observation study alongside the experimental intervention, with the aim of directly identifying the operative mechanisms underlying the potential deterrent effect of body-worn cameras [27]. This qualitative component ensures attention to how organizational culture—including practices of camera use and conflict management—may influence the camera’s effect. [28,29]. Additionally, the field observations offer the possibility to examine inspectors’ emotional labor and discretion, which previous research has highlighted as influential for inspectors and frontline staff [30].

### Objective

The purpose of this study is to test the protective effect of body-worn cameras among ticket inspectors in Denmark. We extend the existing literature, which has focused predominantly on U.S. police officers, by examining this effect in a different national context and occupational group. We build on the study of transport personnel by Ariel and colleagues [4] and address key limitations of previous research by implementing a more robust randomization design at the shift level, collecting survey data on both victimization and perceived safety, and triangulating with field observations to identify situational mechanisms. Through this design, we address a central question in the literature: whether the implementation of body-worn camera in frontline encounters has a preventive effect, and under which conditions.

## Methods

### Study design

The study takes place in collaboration with three traffic companies operating trains, buses, and trams in Denmark to conduct a randomized controlled trial on the preventive effect of body-worn cameras for ticket inspectors. The study builds upon a now finalized pilot study with one of the three companies, where the feasibility of the study design was evaluated. The present paper describes the protocol for the full-scale study in which the intervention’s effectiveness will be tested, noting how the final protocol was informed by insights from a small-scale feasibility pilot.

### Intervention setting

The inclusion of three Danish traffic companies ensures variation in geography, demographics, and organizational contexts, including differences in vehicle types, inspector roles, tasks, and educational backgrounds. The companies also differ in their prior experience with body-worn cameras: one uses them routinely, another participated in the pilot phase of the current study, and the third has never used them. All three companies have in-vehicle surveillance cameras, although in one company these are no longer operational. In parts of the transport sector, body-worn cameras are met with mixed reactions—some inspectors welcome them as a security measure, while others express concern, particularly about surveillance and potential misuse of recordings by management. While the intervention will be implemented in the same overall manner across the three companies, certain elements will be adapted to account for differences in work practices and organizational structures, as outlined below.

Company 1 is Denmark’s largest train operator, employing approximately 850 inspectors who service regional and long-distance routes, as well as trains in the urban area of Copenhagen. The company implemented body-worn cameras on a voluntary basis in April 2023 and aims to broaden the use of cameras among employees, with 200 cameras distributed prior to the intervention and approximately 200 more deployed at its onset. The company participates in the study with the aim of reducing work-related victimization and giving non-wearers the opportunity to become familiar with wearing a camera in practice. The company already has formal guidelines for camera use that include an expectation to activate the camera in incidents where a conflict with a passenger escalates. Inspectors are not obliged to inform passengers that they are recording in case they think activation can escalate the incident. On each camera, there is a small sticker announcing potential recording. Inspectors who work in trains operating across the country work alone, except during evening shift after 10 pm, when they work in pairs for safety reasons. Inspectors patrolling the urban rail system work in pairs during evening shifts after 8 pm.

Company 2 employs approximately 20 inspectors who work on trains serving regional routes. The company is motivated to participate in the study to obtain evidence on the preventive effect of cameras on workplace victimization, which could inform their decision regarding the potential implementation of cameras. Company 2 took part in the pilot study with a subgroup of inspectors, meaning that inspectors have either worn a camera themselves or know colleagues who have. Since the pilot study, the workforce has been reduced by half following a change in the company’s ownership. Inspectors always work alone.

Company 3 operates buses and trains in multiple regions, as well as trams in Aarhus, the second-largest city in Denmark. The company comprises two subcompanies. One is a security company contracted to conduct inspections on buses and trams; from this subcompany, 18 guards are employed to carry out inspections for Company 3. Employees always work in units of two to three. The other subcompany is a traffic company with four inspectors responsible for local train routes in a region. These inspectors always work alone. Neither subcompany uses body-worn cameras during inspections.

### Participants

#### Inclusion criteria and recruitment.

Eligible participants are inspectors employed by one of the participating companies who inspect tickets on trains, trams, or buses in Denmark. Ticket inspectors are recruited voluntarily through the participating companies. Recruitment takes place via informational meetings, organizational newsletters, and field visits to staff depots where inspectors typically pass during their shifts. In these settings, we present the study design, summarize previous research, and outline the expected effects of cameras—while stressing the importance of an intervention study to determine these effects conclusively. Inspectors are encouraged to participate regardless of their personal views on cameras, ensuring more robust findings that can better inform future practice.

In Company 1, recruitment began on June 25, 2025, with the first written consent obtained on July 8, and with the first employees having entered the trial on August 20. In Company 2, recruitment and obtainment of informed consent began on June 11, with the first employees expected to enter the trial on September 18. In Company 3, recruitment and obtainment of informed consent began on August 28, with the first employees expected to enter the trial on September 20. Recruitment across the three companies will run until September 30 and the trial will conclude by December 31, 2025.

Upon recruitment, participants are asked to provide written informed consent to share personal information with the research team, in accordance with the General Data Protection Regulation (GDPR), and to read and sign a participant information sheet to provide written consent to participate in the study. The participant information sheet emphasizes that participation is voluntary and acknowledges that the study may involve some risk. In theory, wearing a camera could increase exposure to aggression, although the study anticipates the opposite—a protective effect. Highlighting this potential risk is especially important for employees of Company 1, who already wear cameras in their daily work but will be required to remove the device during control shifts as part of the experiment. The participant information sheet also provides guidance on wearing and using the camera in a safe and de-escalatory manner. Finally, it informs participants that any criticism or concerns they share with the research team, or their decision to opt out, will be treated confidentially to avoid any repercussions from management. Prior to starting in the trial, each participant is asked either over phone or in person to confirm that they still consent to participate in the study.

### Randomization

#### Camera use.

The study randomizes at the level of inspector shifts. Each inspector is assigned to wear a camera during a randomly selected 50% of their shifts over the study period (treatment) and to work without a camera during the remaining shifts (control). Four hours before each shift, participants receive an automated text message indicating whether they must wear a camera for that shift. If a shift starts before 12 pm, the text message is sent the evening before at 8 pm to avoid contacting participants between 8 pm and 8 am. The treatment message is sent within a time span that allows participants to have time to bring the camera to work while minimizing the risk that participants select routes based on whether they carry the camera or not (as some inspectors have freedom in choosing routes). While this design informs inspectors about their condition assignment in a timely manner, it does not eliminate the risk that inspectors adjust their inspection style and manner of coping with passenger conflicts in anticipation of their assigned condition. To assess this bias, we will examine whether outcomes vary across different notification times, on the assumption that shorter notice leaves less room for anticipatory adjustments.

The block-randomization design guarantees an even 50/50 split between treatment and control shifts for every participating inspector. Compared to the field trial by Ariel and colleagues [4], this approach offers key advantages: it ensures treatment and control observations are independent of inspector- or shift-specific characteristics, removes clustering by inspector, increases statistical power, and makes it more likely that randomization produces genuine *ceteris paribus* conditions [31,32]. Despite these strengths, this design carries a risk of contamination, as the same inspectors alternate between treatment and control shifts and may carry learned behaviors, fatigue effects, or other carry-over biases from one condition to the other [see, e.g., 33].

#### Team-level.

Inspectors from Company 1 occasionally and Company 3 always work in pairs, potentially causing spillover effects from the expected benefits of wearing a camera to colleagues. This is accounted for in the survey by asking whether the inspector worked alone or together with a colleague during the shift. For inspectors from the subcompany hired by Company 3 who always work with two or three colleagues, the randomization and treatment are allocated at the dyad-team-level to prevent spillover effects.

#### Instruction.

Implementing body-worn cameras requires clear instructions on their use, including when to activate them and how to inform passengers. Company 1 already has established routines, so staff will continue current practices. Companies 2 and 3 lack such routines; to ensure comparable implementation, they will adopt instructions similar to Company 1’s. However, some differences between companies are to be expected, particularly regarding the extent to which the presence of the cameras is announced to passengers. Since treatment fidelity regarding specific camera-use practices cannot be fully enforced across organizational contexts, we emphasize that the study’s strongest test is of the effect of the mere presence of a body-worn camera on the inspector’s uniform—the consistent element across all conditions—rather than how the camera is operated with slight differences between companies. As per the instructions, activation of the camera relies on the inspector’s assessment of a given situation, meaning that the camera only records when the inspector decides to record an incident.

### Camera visibility

In our pilot study, which included field observations of inspections *in situ*, we found that most passengers did not notice the camera on the inspector’s uniform during normal inspections. This may explain the lack of effect observed in prior studies: if passengers are unaware of the camera, it cannot influence their behavior. To investigate this further, we added a camera-with-badge condition to the experiment. In this condition, inspectors wear a clearly visible badge next to the camera, informing passengers that they may be recorded. The experiment therefore includes two treatment conditions—camera without badge and camera with badge—and one control condition without a camera or badge. The camera-with-badge condition is not applied in Company 1, as it already follows established camera-use practices that do not involve a badge. Thus, this three-arm design component is only examined in analyses excluding Company 1. The main two-arm analysis—comparing camera versus no camera—will be based on pooled data from all three companies.

### Survey

#### Baseline and background survey.

Before the intervention, inspectors complete a questionnaire assessing baseline self-perceived safety, attitudes toward body-worn cameras, trust in management, and sociodemographic information such as age, gender, ethnicity, and height and weight (for calculating body mass index, BMI). These data enable the examination of potential treatment effect heterogeneities—for example, whether women or individuals with lower BMIs experience greater benefits from body-worn cameras than colleagues with a more imposing physical presence. Identifying such heterogeneities will enhance the generalizability of the findings to other companies and contexts, providing more nuanced insights than a simple average treatment effect.

#### Shift survey.

At the end of each shift, inspectors receive an automated text message prompting them to complete a short questionnaire. This survey captures self-reported experiences of victimization (ranging from verbal condescension to physical aggression and violence), perceived safety, whether they worked with a colleague, and whether they wore a camera. The latter information enables estimation of the local average treatment effect among inspectors who comply with their randomly assigned treatment, using instrumental variable regression [34]. In the remainder of the paper, we refer to this questionnaire as “the survey.”

### Measures

#### Outcome measures.

The primary outcome measure is victimization, ranging from verbal offenses and harassment to physical violence. In the survey, participants report whether they experienced each of the following passenger behaviors 0, 1, 2, 3 or more times during the shift: behaved unpleasantly toward the inspector; were rude or condescending; raised their voice or cursed at the inspector; filmed the inspector with their phone against their will; came uncomfortably close in a threatening manner; touched or grabbed the inspector in an unpleasant way; acted aggressively or threateningly toward the inspector (e.g., banging on the window or threatening with an object); made verbal threats; spat at the inspector; or used physical violence (e.g., pushed, hit, or threw an object at the inspector). We will use Mokken scale methods to combine these observed measures into a single victimization scale and assess its measurement quality.

The victimization survey instrument is developed based on previous research on passenger aggression [35,36] and on Company 1’s incident records, which document the specific behaviors that occur in the registered victimization events. By grounding our measure in these context-specific descriptions rather than in abstract theoretical categories or instruments developed for other settings, we enhance the construct validity of our victimization measure.

The secondary outcome measure is self-perceived safety, based on inspectors’ assessments of how safe they felt during their most recent shift. It is measured on a five-point Likert scale ranging from not at all (1), to a low extent (2), to some extent (3), to a high extent (4), and to a very high extent (5), using a set of questions evaluating the extent to which the inspector felt unsafe, feared being assaulted, and avoided a passenger for their own safety during the shift. These items will be combined into an additive scale.

The primary and secondary outcome measures are based on inspectors’ subjective ratings of encountered passenger behavior and self-perceived safety during a shift. This reliance on self-report comes with the risk of expectancy bias, as inspectors may rate their experiences in accordance with treatment assignment—or conversely, may underreport benefits given some employees’ skepticism toward body-worn cameras. Blinding is not feasible, as inspectors are necessarily aware of whether they are wearing a camera—a common type of limitation in experimental social science research [37]. However, the parallel field observations provide some qualitative triangulation by offering independent insight into how staff-passenger encounters unfold.

#### Moderators.

We include a variety of moderators to explore heterogeneities in the camera’s effect on victimization. One group of moderators is measured in the initial baseline survey and includes the inspector’s gender, age, ethnicity, household status, work experience in the company, BMI, prior victimization, prior sick leave due to victimization, baseline sense of safety, and attitudes toward cameras and management. Another group of moderators includes company, shift start and end time, whether the inspector worked alone or in units for some or most of the shift, camera placement on the uniform, and whether a colleague was wearing a camera. All of these moderators are pre-treatment variables and are voluntarily provided by participants.

### Data analyses

Our data analysis plan proceeds in three stages to comprehensively evaluate the impact of body-worn cameras. We will estimate the causal effect for two primary continuous outcomes: victimization and perceived safety. First, we will estimate the Intention-to-Treat (ITT) effect, which is the effect of the random *assignment* to carry a camera, regardless of actual use. This analysis preserves the benefits of the initial randomization. We will use an Ordinary Least Squares (OLS) regression model for this purpose:


$$Y_{i}=\beta_{\text{0}}+\;\beta_{\text{1}}Z_{i}+\epsilon_{i}$$

Here, Y_i is the outcome for inspector i, and Zi is a treatment indicator equal to 1 if the inspector was assigned to the camera group and 0 otherwise. The coefficient of interest, β1, represents the ITT effect.

Second, to account for potential non-compliance (i.e., inspectors not using their assigned cameras), we will estimate the Average Treatment Effect on the Treated (ATT). This represents the effect of actually using a camera for those who complied with the assignment. We will use the random assignment as an instrumental variable (IV) for reported camera use. The effect will be estimated using a Two-Stage Least Squares (2SLS) regression.

First Stage: Predict actual camera use based on the random assignment. Here, Di is an indicator for whether inspector i reported using the camera.


$$D_{i}=\gamma_{\text{0}}+\;\gamma_{\text{1}}Z_{i}+\nu_{i}$$

Second Stage: Use the predicted camera use from the first stage (D^i) to estimate the effect on the outcome. The coefficient δATT provides our estimate of the ATT.


$$Y_{i}=\lambda_{\text{0}}+\;\lambda_{\text{ATT}}{\hat{D}}_{i}+\eta_{i}$$

Finally, to explore potential moderators, we will investigate treatment effect heterogeneity. We will use causal forests, a machine-learning method designed to estimate Conditional Average Treatment Effects (CATEs), defined as τ(X)=E[Y(1)−Y(0)|X = x]. This non-parametric approach will allow us to flexibly explore how the treatment effect varies across a range of pre-specified inspector characteristics (X) without being constrained by linear interaction terms. These explorative subgroup analyses will be conducted and disseminated with careful consideration of sensitive moderator variables such as BMI and ethnicity. Further, we acknowledge that our exploratory subgroup analyses imply an increased false-positive rate due to multiple comparisons [38], which will be addressed by interpreting statistical significance tests conservatively and clearly labeling these results as’exploratory’ in the Results section.

For all OLS and 2SLS regression models, we will calculate cluster-robust standard errors to account for the statistical dependence between inspectors working in dyads. Furthermore, as a robustness check, we will re-estimate our primary models including inspector fixed effects to control for any unobserved, time-invariant characteristics of the individuals.

### Sample size and power analysis

The current study will sample approximately 3,000 shifts from up to 60 ticket inspectors employed by the three companies during a three to four months intervention period. As mentioned, our block-randomization design using work shifts, not individual inspectors, ensures that treatment and control observations are independent of inspector characteristics, effectively making the 3,000 shifts the sample unit [32].

Importantly, the sample size in the current study will provide very high statistical power to detect even small effects. A priori power analysis indicates that the planned sample size will yield 90% power to detect very small effect sizes (Cohen’s *f*^2^ = 0.003) in a regression model. Even under conservative assumptions of substantial attrition—such as 20% missing data due to loss of interest among inspectors who fail to complete the survey—the study is still expected to maintain 90% power to detect similarly small effects (Cohen’s *f*^2^ = 0.004).

Further data-structural and model-specification choices may reduce this power. For example, this could result from the use of cluster-robust standard errors to account for inspectors working in dyads, the imbalance in the number of observations across experimental conditions, or other anomalies that may arise during data collection. Nevertheless, given that our power calculations indicate the ability to detect even very small effects, it is unlikely that such factors will meaningfully compromise the overall capacity of the analyses to detect effects of a small magnitude.

### Field observations

In parallel with the rollout of the experiment, we will conduct field observations of inspectors’ work to gain an in-depth understanding of how cameras influence interactions between inspectors and passengers in practice. The primary aim is to explore the situated mechanisms that lead camera use either to have an effect or to have no effect. The fieldwork will involve one to two trained research assistants accompanying inspectors during their workdays. The number of observation days will be a minimum of 10 workdays, but the final total will be determined as the fieldwork progresses, depending on whether additional days yield new insights.

### Pilot feasibility study

Following good research practice for intervention studies [39], a pilot study was conducted in summer 2023 to explore the feasibility of testing the effect of body-worn cameras. The 20-day pilot tested block-randomizing body-worn camera-use across 83 work shifts for ten ticket inspectors employed by Company 3. To gain qualitative insight into inspectors’ attitudes and perceptions about cameras and the situational aspects of camera use, we also interviewed inspectors before the piloting phase and conducted field observations over two workdays where the inspectors wore cameras.

The pilot results showed that the randomized controlled trial is practically feasible, with minimal attrition and a well-functioning questionnaire capable of capturing even more subtle forms of victimization. Further, qualitative observations and interview material indicated that few passengers noticed the cameras, suggesting that further work is needed to test the role of camera visibility. See data from the pilot study at osf.io/tk5xq/.

### Ethics approval and consent

Intervention studies raise ethical questions, especially when the manipulation involves a principal risk that the individuals involved may be exposed to risks [37]. The study was approved by the Aalborg University Research Ethics Committee (Case No.: 2025-505-00488), and the pilot study was approved by the Department of Sociology’s Ethics Review Board at the University of Copenhagen (Case No.: 2022-0-2022-05).

Ethical procedures include voluntary participation based on written informed consent. As previously mentioned, the participant information sheet contains details on the study’s aim and expectation, the hypothetical risk of camera use, the purpose of personal data collection, anonymity, and an opt-out option without notification of the participant’s employer. Furthermore, the procedures involve obtaining informed consent about sharing personal information, providing the research team’s contact details to allow participants to raise concerns, and implementing a process to exclude participants who repeatedly express such concerns. A member of the project group must confirm that a participant has received the intended information about the study with a signature on each of the participant information sheets.

### Inclusivity in global research

Additional information regarding the ethical, cultural, and scientific considerations specific to inclusivity in global research is included in the Supporting Information File.

### Data management

All collected data will be stored securely in accordance with Aalborg University’s and the University of Copenhagen’s security regulations. Once data collection is complete, analyses will be conducted on a pseudonymized dataset without participants’ names to minimize unnecessary personal identification. The non-anonymized data will be stored separately on a secure server and accessed only if needed for data validation during the analysis phase. Upon project completion, all data will be fully anonymized to ensure the confidentiality of participants.

## Discussion

The adoption of body-worn cameras reflects two contrasting trends: growing implementation by organizations employing frontline workers, and a comparatively fragile evidence base underpinning this practice. This study aims to help close this gap by examining the preventive effect of body-worn cameras in the transportation sector, which—like many other frontline sectors—is experiencing rapid growth in the use of body-worn cameras as a safety technology. Specifically, by sampling Danish transportation inspectors, the study addresses the need for research conducted in contexts other than U.S. policing.

The study presents a robust experimental design with block-randomization at the shift level and survey-based measures of both perceived safety and victimization. The victimization measure spans from physical violence to verbal abuse and harassment—a common experience among transport staff but rarely captured in prior research, which has relied on official incident records. Additionally, we advance the literature by conducting a parallel field observational study, which sheds light on the situational mechanisms through which cameras may influence staff-passenger interactions.

Despite these strengths, limitations remain. First, camera practices vary across the three included companies, which may affect the comparability of treatment conditions. Second, our reliance on outcome measures collected through self-report from unblinded inspectors introduces a risk of expectancy bias, with inspectors potentially over- or underreporting victimization depending on their attitudes toward cameras. Third, the design does not safeguard against inspectors adapting their behavior based on treatment assignment or carrying learned behaviors from one condition to the other.

Accordingly, our study—like any single study—cannot definitively settle the question of whether body-worn cameras have a protective effect, underscoring the need for further replications and meta-analytical syntheses to evaluate the robustness and heterogeneity of effects across diverse contexts.

## Supporting information

S1 File Inclusivity in global research questionnaire. (DOCX)
